# Histopathologic Parameters at Diagnosis as Early Predictors of Histologic Remission along the Course of Ulcerative Colitis

**DOI:** 10.1155/2020/8891937

**Published:** 2020-12-03

**Authors:** Jesús K. Yamamoto-Furusho, Braulio Martínez-Benítez, Germán E. Sánchez-Morales

**Affiliations:** ^1^Inflammatory Bowel Disease Clinic, Department of Gastroenterology, Instituto Nacional de Ciencias Médicas y Nutrición Salvador Zubiran, Mexico City, Mexico; ^2^Department of Pathology, Instituto Nacional de Ciencias Médicas y Nutrición Salvador Zubiran, Mexico City, Mexico

## Abstract

**Background:**

Currently, the treatment goal in ulcerative colitis (UC) is to achieve clinical and endoscopic remission; nevertheless, histologic remission is a potential new treatment goal since it is associated with favorable long-term clinical outcome lower rates of hospitalization, complications, and colectomies.

**Aim:**

Evaluate clinical and histopathologic characteristics at diagnosis as potential predictors of histologic remission in patients with ulcerative colitis.

**Methods:**

This is a retrospective cohort study from 2007 to 2014, including 260 patients. Clinical and demographic information and Mayo endoscopic and Riley histologic grade were obtained accordingly with the follow-up. Histological evaluation was made for all 260 patients; fifty-six patients with histologic remission at the follow-up underwent separate evaluation of mucosal biopsy at the moment of diagnosis. Univariate and multivariate analyses were applied to data from these 56 patients to identify histologic features at diagnosis associated with histologic remission during follow-up. The odds ratio (OR) was determined as a measure for the strength of association. A *P* value of less than 0.05 was taken as a level of significance.

**Results:**

The frequency of histologic remission according to the Riley index in our study group was 21.5%. Factors associated with histologic remission were treatment with steroids (*P* = 0.01, OR = 0.38, CI 95% = 0.16‐0.90), reduced mucin production (*P* = 0.02, OR = 0.23, CI 95% = 0.06–0.86), and less than 10 eosinophils per high power field (*P* = 0.001, OR = 6.66, CI 95% = 2.03–21.84).

**Conclusion:**

Factors that impair histologic remission in patients with ulcerative colitis were treatment with steroids and reduced mucin production; meanwhile, less than 10 eosinophils per high power showed a predictive value for histologic remission.

## 1. Introduction

Ulcerative colitis (UC) is a chronic inflammation of the colonic mucosa characterized by periods of clinical activity and remission; it is a worldwide disorder with geographic heterogeneity and multifactorial etiology [1]. Currently, UC's treatment goal is to achieve clinical and endoscopic remission, understanding clinical remission as baseline bowel function with no bleed in feces, and endoscopic remission as normal mucosa with or without visible vascular pattern or erythema but with no bleeding. Those treatment targets are linked with a lower rate of hospitalization, complications, and colectomies [2]. Nevertheless, recent studies have proposed histologic remission, defined as mucosal healing and resolution of the crypt architectural distortion and inflammatory infiltrate [3], as a possible new treatment goal due to its association with even better long-term clinical outcomes like a lower risk of relapses, reduced risk of colorectal cancer, and decreased rate of hospitalization and surgery, altogether improving the quality of life in patients with UC [4].

Several studies have shown that patients without any treatment have a clinical relapse rate of 58–89%, showing the disease's changing activity nature; in the scenario where treatment is given, the relapse rate decreases to 12-50% [5]. According to Baar's study, from those patients with clinical remission, 37% had endoscopic and histologic activity; meanwhile, 31% had endoscopic remission with histologic activity. Furthermore, from those patients with endoscopic remission, 26% had histologic remission, 26% mild histologic activity, and 7% moderate histologic activity, demonstrating that mucosal healing is not always an indicator of histologic healing. In that same study was observed that histologic activity is associated with 61% of endoscopic relapse. Throughout the follow-up, those patients with histologic activity and endoscopic remission seem to have a similar prognosis than those with endoscopic activity. [6]

Clinical characteristics like younger age at diagnosis (*P* < 0.03), shorter remission period (*P* < 0.03), and a greater number of relapses in female patients (*P* < 0.03) increase the risk of the clinical and endoscopic activity, although the latter is controversial in some studies; moreover, few serological parameters like p-ANCA titers (P 0.002) and total ANCA titer (*P* < 0.03) have shown an association with the risk of relapse; however, their instability through time as a serological marker makes it difficult to use them to guide treatment effort [5].

From a histologic point of view, active inflammation is characterized by damage to the mucosal epithelium, usually by neutrophils that generate crypt abscess, crypt destruction, erosion, and ulceration [7]. These histologic features that have shown an association with clinical relapse include increased basal eosinophils, increased basal neutrophils, increased neutrophils in the epithelium, excess of neutrophils in crypts, and the presence of basal plasmacytosis; meanwhile, crypt distortion and chronic inflammation infiltrate appear to have no role in the risk of relapse [8].

Many efforts have been focused on determining factors that can identify patients at risk of relapse to ensure close follow-up and proper treatment optimization, preventing future complications. Still, there is little information about those factors that could predict histologic remission and identify patients who might require a more flexible follow-up and even treatment withdrawal.

## 2. Material and Methods

### 2.1. Patients

Two hundred and sixty patients with the diagnosis of UC confirmed by histology were studied. All patients with CUCI belonged to the Inflammatory Bowel Diseases Clinic at the National Institute of Medical Sciences and Nutrition in Mexico City between January 1st, 2007, and December 31th, 2014. Histological evaluation was made for all 260 patient; fifty-six patients with histologic remission at the follow-up underwent separate evaluation of mucosal biopsy at the moment of diagnosis. Univariate and multivariate analyses were applied to data at diagnosis between 56 patients with histologic remission during follow-up and 56 patients without histology remission during follow-up chosen randomly. The aim of this work is to identify histologic features at diagnosis associated with histologic remission during follow-up in patients with CUCI.

Together with Mayo endoscopic and Riley histologic grades, demographic and clinical data were gathered from medical records and direct interviews. Variables evaluated were gender, age at diagnosis, family history of inflammatory bowel disease (IBD), extraintestinal manifestation (EIM), concomitant autoimmune disease, histologic activity, elevated C-reactive protein (CRP), p-ANCAs, an extension of the disease, clinical course, and treatment.

A cutoff level of 0.36 mg/dl for hs-CRP was considered high since a previous study from Yamamoto-Furusho and colleges showed a good correlation with histological activity [9]. The clinical course of the disease was defined as active with posterior long remission (first episode followed by a remission for more than 5 years), intermittent (fewer of 2 relapses per year), and chronic activity (persistent activity even with medical treatment).

### 2.2. Histologic Evaluation

Fifty-six patients from the same cohort with histologic remission at the follow-up underwent separate exhaustive evaluation of mucosal biopsy at the moment of diagnosis. Samples evaluated were from mucosa biopsies at the terminal ileum, ascending, transverse, descending, sigmoid, and rectum and obtained throughout ileocolonoscopy according to international guidelines. Samples were fixed with 10% formalin for hematoxylin and eosin staining. Histopathologic parameters evaluated were basal plasmacytosis, lamina propria irregularities, polymorphonuclear leukocytes in the lamina propria, crypt abscesses, crypt abscesses rupture, glandular distortion, glandular regenerative changes, reduced mucin production, and eosinophils in the lamina propria. When there were parameters with different grades along the colon, the highest scores were considered for statistical purposes. Patients with histologic remission may remain with structural changes, even in the absents of acute inflammation, like crypt distortion and mucosal atrophy; therefore, a general histologic evaluation was score according to the Riley index, where Riley 0-1 were considered histologic remission.

### 2.3. Statistical Analysis

Frequency and proportions were included for qualitative variables. The chi-squared test was used to analyze categorical variables. Student's *t*-test or Mann–Whitney *U* test was applied for independent samples accordingly to variable data distribution. Wilcoxon or Friedman test was used for related samples. Those risk factors with *P* values < 0.1 in the univariate analysis were included in the multivariate regression model. The results were expressed as odds ratio (OR) with a corresponding 95% confidence interval (CI). Two-tailed significance tests were used in all statistical analyses, and *P* < 0.05 was considered statistically significant. Information analysis was made with SSPS Version 21.

## 3. Results

This study included 260 patients with a confirmed diagnosis of UC, 125 females (48.10%) and 135 males (51.90%) with a mean diagnosis age of 31.6 years old (range 6 to 65 years old). The achievement of histologic remission was observed in 56 patients (21.5%). The disease's extent was distributed as follows: pancolitis in 78.5%, left colitis in 5%, and proctosigmoiditis in 16.5%. Regarding the clinical course, 13.5% had an active then inactive course, 77.7% had an intermittent activity, and 8.8% had chronic continual activity. Extraintestinal manifestations were present in 145 patients (56.0%). The hs-CRP was elevated in 127 persons (48.84%), and 86 individuals (33.1%) were positive for p-ANCAs. Concerning the medical treatment, the distribution was as follows: 5–ASA (aminosalicylic acid) 91.9%, steroids 23.8%, azathioprine 29.2%, anti-TNF (antitumor necrosis factor) 3.1%, and colectomy 3.80%, as shown in Table 1.

In the initial univariate analysis, clinical factors associated with histologic remission in a patient with UC were p-ANCAs positivity (*P* = 0.05), left colitis (*P* = 0.03), and steroids treatment (*P* = 0.01); however, after multivariate analysis, steroid treatment (*P* = 0.03, OR = 0.38, 95%IC = 0.16–0.90) was the only one variable that remains statistically significant as shown in Table 2.

Table 3 shows a resume of all characteristics evaluated and their frequency in the fifty-six patients assessed regarding histologic evaluation. Features that showed relation with histologic remission in the univariate analysis included polymorphonuclear leukocytes in the lamina propria grade 0 (*P* = 0.03), polymorphonuclear leukocytes in the lamina propria grade 3 (*P* = 0.05), glandular distortion grade 0 (*P* = 0.04), reduced mucin production grade 2 (*P* = 0.02), and eosinophils in the lamina propria less than 10 per high power field (0.001). After multivariate analysis, reduced mucin production grade 2 (*P* = 0.03, OR = 0.23, 95%IC = 0.06 − 0.86) and eosinophils in the lamina propria less than 10 per high power field (*P* = 0.01, OR = 6.66, 95% IC 2.03-21.84) remain statistically significant as shown in Table 4.

## 4. Discussion

Ulcerative colitis is a disease characterized by the presence of chronic inflammation associated with remission and relapsing activity periods. Currently, the goal treatment is to achieve mucosal healing documented by endoscopy as a mean to decrease the incidence of long-term complications like colorectal cancer and improve quality of life; however, even with documented endoscopic remission, there is a 30-40% of patient with microscopic evidence of inflammation [10] that remain at risk of hospitalization and surgery.

Many of the reported studies in the literature are focused on identified risk factors related to early relapse. Nowadays, there is well established histologic factor associated with future activity like the grade of neutrophils infiltrate [11], mucin depletion [12], basal plasmacytosis [13], crypt abscesses [14], and cryptitis [15]; however, there is little information about histologic factor linked to a more benign clinical course.

This study's main objective was to identify early clinical and histologic features associated with histologic remission in a patient with UC to recognize those potential patients that could be benefited from a more flexible treatment and follow-up. On univariate analysis, we found that those clinical variables that impair histologic remission were p-ANCA positivity, presence of left colitis, steroid, and azathioprine treatment. Nevertheless, after multivariate analysis, steroid treatment was the only feature that persists statistically significant. This finding may represent a subgroup of patients whose clinical course demands continuous or frequent use of steroids, thus a subgroup of patients where histologic remission is harder to achieve.

Concerning histologic characteristics, greater polymorphonuclear leukocytes in the lamina propria, greater glandular distortion, and reduced mucin production were variables that impaired histologic remission on univariate analysis. Besides, an infiltrate of eosinophils in the lamina propria less than 10 per high power field was associated with microscopic remission along the course of UC. After the multivariate analysis, the presence of less than 10 eosinophils per high power field in the lamina propria remained statistically significant as a factor of histologic remission, suggesting the important role of eosinophils in the mucosal inflammation in UC patients. It is thought that chemotactic factors and alteration in the mucosal barrier are the mechanisms throughout eosinophils that participate in tissue injury [16–19]; thus, it might be assumed from this work that patients with low eosinophilic infiltrate in mucosal biopsies could maintain relative mucosal integrity that allows histologic remission.

In conclusion, factors that impair the histologic remission in patients with ulcerative colitis were treatment with steroids and reduced mucin production; meanwhile, less than 10 eosinophils per high power showed a predictive value for histologic remission.

## Figures and Tables

**Table 1:** Clinical and demographic characteristics of patients with ulcerative colitis (*n* = 260).

Characteristics	*n*	%
Gender		
Female	125	48.1
Male	135	51.9
Age at diagnosis		
Before 17 years old	18	6.9
Between 17–40 years old	181	69.6
After 40 years old	61	23.5
Family history of IBD	6	2.30
Extraintestinal manifestations	145	56.0
Concomitant autoimmune disease	24	9.2
Histologic remission	56	21.5
Elevated hs-CRP (>0.36 mg/dl)	127	48.8
Positive p-ANCAs	86	33.1
Extension of the disease		
Pancolitis	204	78.5
Left colitis	13	5.0
Proctosigmoiditis	43	16.5
Clinical course		
Active then inactive	35	13.5
Intermittent activity	202	77.7
Chronic activity	23	8.8
Treatment		
5–ASA (oral or topical)	234	91.9
Steroids	62	23.8
Azathioprine	76	29.2
Anti-TNF	8	3.1

IBD: inflammatory bowel disease; hs-CRP: high sensitive C-reactive protein; ANCAs: antineutrophil cytoplasmic antibody; 5-ASA: 5-aminosalicylic acid.

**Table 2:** Univariate and multivariate analysis for clinical characteristics at diagnosis associated with histologic remission during follow-up in patients with ulcerative colitis.

Variable	Univariate	Multivariate	OR	95% CI	*r*	*r*2
	*P* value	*P* value				
Age at diagnosis						
Before 17 years old	0.06	0.06	2.50	0.92-6.80	0.12	0.01
Between 17–40 years old	0.07	0.10	0.59	0.32-1.11	-0.10	0.01
Positive p-ANCAs	0.05	0.08	0.54	0.27-1.07	-0.11	0.01
Extension of the disease						
Left colitis	0.03	0.09	0.26	0.03-2.04	-0.12	0.01
Treatment						
Steroids^∗^	0.01	0.03	0.38	0.16-0.90	-0.14	0.02
Azathioprine	0.09	0.15	0.59	0.29-1.20	-0.09	0.01

**Table 3:** Histologic characteristics of mucosal biopsies at diagnosis of ulcerative colitis in patients with documented remission through their clinical course.

	Remission		No remission	
	*n*	%	*n*	%
Basal plasmacytosis	30	100	26	100
Grade 0	0	0	2	8
Grade 1	3	10	5	19
Grade 2	18	60	13	50
Grade 3	9	30	6	23
Lamina propria irregularities	30	100	26	100
Grade 0	3	10	6	23
Grade 1	5	17	9	35
Grade 2	11	37	5	19
Grade 3	11	37	6	23
Polymorphonuclear leukocytes in the lamina propria	30	100	26	100
Grade 0	2	7	8	31
Grade 1	8	27	6	23
Grade 2	11	37	10	38
Grade 3	9	30	2	8
Crypt abscesses	30	100	26	100
Grade 0	17	57	15	58
Grade 1	9	30	7	27
Grade 2	3	10	2	8
Grade 3	1	3	2	8
Crypt abscesses rupture	30	100	26	100
Grade 0	13	43	15	58
Grade 1	9	30	6	23
Grade 2	7	23	4	15
Grade 3	1	3	1	4
Glandular distortion	30	100	26	100
Grade 0	0	0	4	15
Grade 1	6	20	6	23
Grade 2	17	57	8	31
Grade 3	7	23	8	31
Glandular regenerative changes	30	100	26	100
Grade 0	2	7	5	19
Grade 1	9	30	8	31
Grade 2	16	53	10	38
Grade 3	3	10	3	12
Reduced mucin production	30	100	26	100
Grade 0	2	7	9	35
Grade 1	10	33	10	38
Grade 2	13	43	4	15
Grade 3	5	17	3	12
Eosinophils in the lamina propria	30	100	26	100
<10 per high power field	10	33	20	77
11-20 per high power field	12	40	5	19
21-30 per high power field	6	20	1	4
31-40 per high power field	2	7	0	0

**Table 4:** Univariate and multivariate analysis for pathologic characteristics at diagnosis associated with histologic remission during follow-up in patients with ulcerative colitis.

Variable	Univariate	Multivariate	OR	95% CI	*r*	*r*2
	*P* value	*P* value				
Polymorphonuclear leukocytes in the lamina propria grade 0	0.03	0.13	6.22	1.18-32.68	0.31	0.10
Polymorphonuclear leukocytes in the lamina propria grade 3	0.05	0.56	0.19	0.03-1.00	-0.28	0.08
Glandular distortion grade 0	0.04	0.27	5.27	0.55-50.5	0.29	0.08
Glandular distortion grade 2	0.06	0.54	0.54	0.18-1.58	-0.26	0.07
Reduced mucin production grade 2^∗^	0.02	0.03	0.23	0.06-0.86	-0.30	0.09
Eosinophils in the lamina propria less than 10 per high power field^∗^	≤0.001	0.01	6.66	2.03-21.84	0.43	0.18

## Data Availability

The data used to support the findings of this study are included within the article.
